# Supplementary material for: Abnormally high exertional breathlessness predicts mortality in people referred for incremental cycle exercise testing
Source: PLoS One. 2024 Dec 18;19(12):e0302111. doi: 10.1371/journal.pone.0302111 (PMC11654960; doi:10.1371/journal.pone.0302111)

**Abnormally high exertional breathlessness predicts mortality in people referred for incremental cycle exercise testing**

**S1. Fig.** Flowchart illustrating the number and percent of a) females and b) males who had normal (white), abnormally high (yellow) or missing (grey) exertional breathlessness intensity ratings (Borg CR10 scale) at any given power output (W) as % of the subject’s predicted maximal power output (%predW_max_). The figure is divided by the different %predWmax intervals, starting with the 25% level. Normality or abnormality of breathlessness on each exercise phase is shown.

a) Females


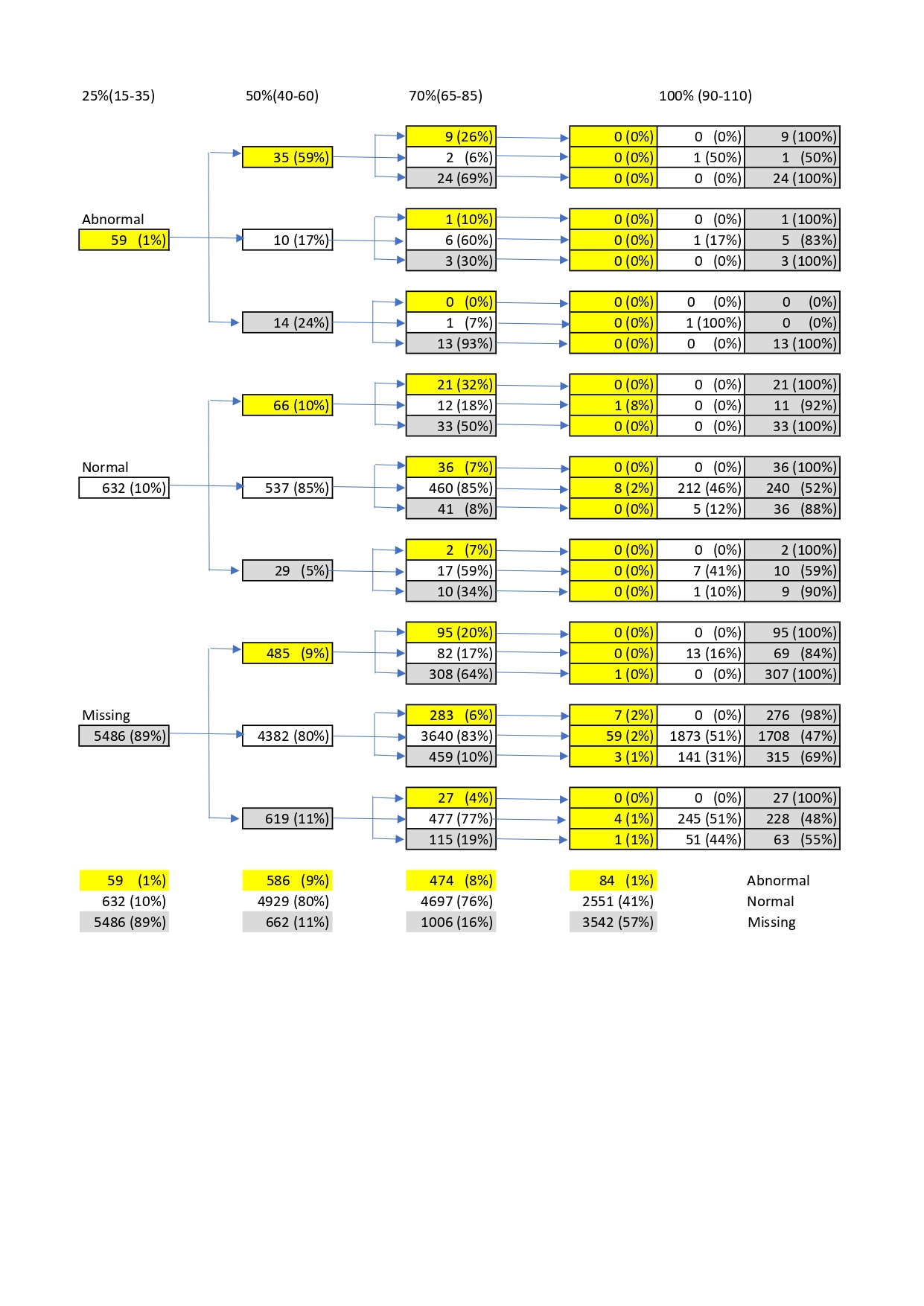


b) Males


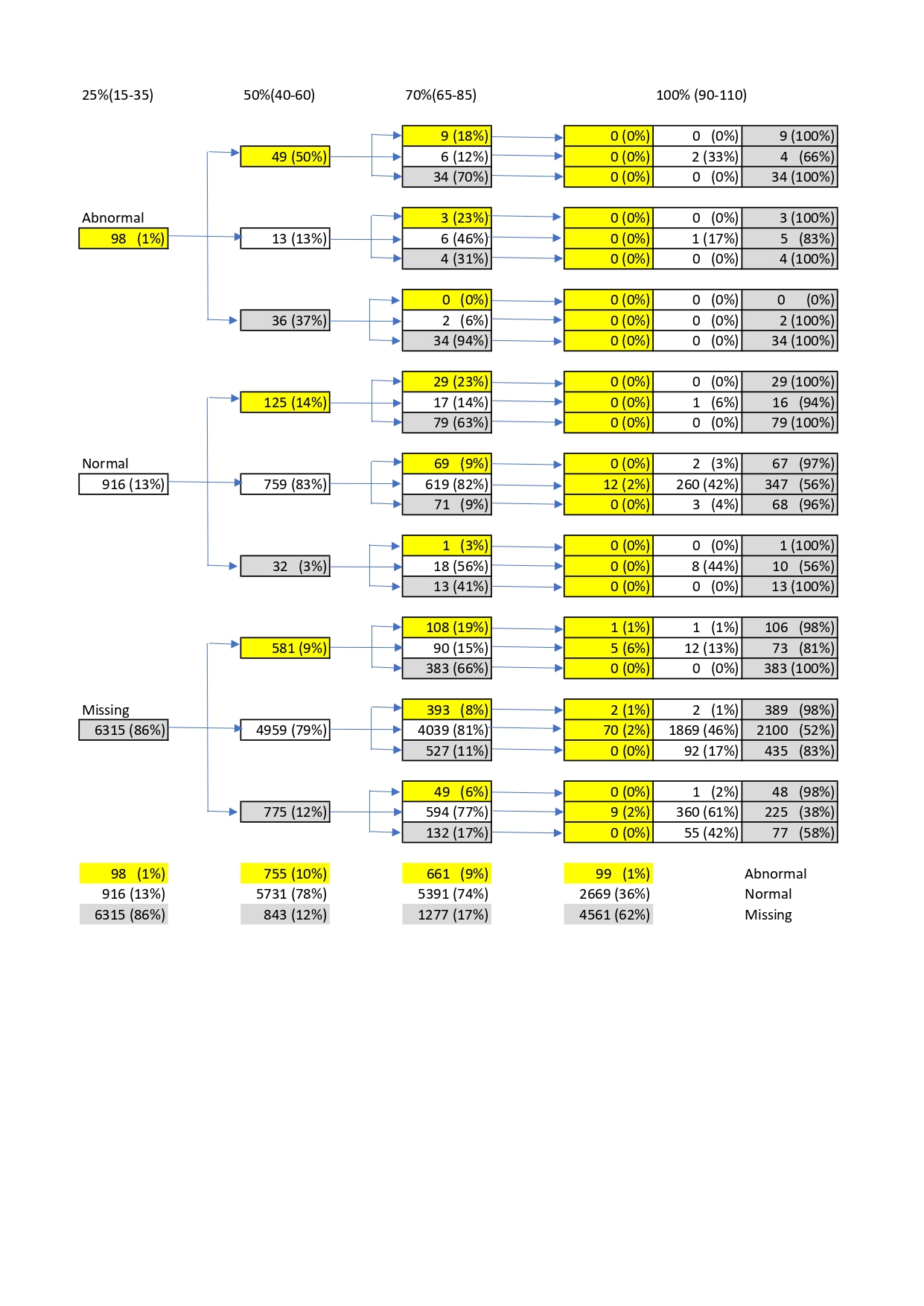

Supplement: S1 Fig — The figure is divided by the different %predWmax intervals, starting with the 25% level. Normality or abnormality of breathlessness on each exercise phase is shown. (DOCX) [file pone.0302111.s001.docx]
